# Giant Cell Tumor of the Pes Anserine Bursa (Extra-Articular Pigmented Villonodular Bursitis): A Case Report and Review of the Literature

**DOI:** 10.1155/2011/491470

**Published:** 2011-06-01

**Authors:** Haitao Zhao, Aditya V. Maheshwari, Dhruv Kumar, Martin M. Malawer

**Affiliations:** ^1^Department of Orthopedic Oncology, Beijing JiShui Tan Hospital, Peking University, 31 Xinjiekou East Street, Xicheng District, Beijing 100035, China; ^2^Department of Orthopedic Oncology, Washington Hospital Center, 110 Irving Street North West, Washington, DC 20010, USA; ^3^Department of Orthopedic Surgery, SUNY Downstate Medical Center, 450 Clarkson Avenue, P.O. Box 30, Brooklyn, NY 11203, USA; ^4^Department of Pathology, Washington Hospital Center, 110 Irving Street North West, Washington, DC 20010, USA; ^5^Department of Orthopedic Oncology, The George Washington University Hospital, 900 23rd Street North West, Washington, DC 20037, USA

## Abstract

Pigmented villonodular synovitis (PVNS) is a rare, benign, proliferating disease affecting the synovium of joints, bursae, and tendon sheaths. Involvement of bursa (PVNB, pigmented villonodular bursitis) is the least common, and only few cases of exclusively extra-articular PVNB of the pes anserinus bursa have been reported so far. We report a case of extra-articular pes anserine PVNB along with a review of the literature. The lesion presented as a painful soft tissue mass in the medial part of the proximal leg. A magnetic resonance imaging showed areas of low to intermediate signals in all sequences and the lesion enhanced heterogeneously with contrast. Diagnosis was confirmed by an incisional biopsy, and an intralesional resection was performed. The postoperative course was uneventful, and the patient is free of disease with no functional deficit at 2 years followup. As with other rare lesions, clinical and radiographic findings in addition to histological examination are essential for correct diagnosis.

## 1. Introduction

Pigmented villonodular synovitis (PVNS) is a rare, benign, proliferating disease affecting the synovium of joints, bursae, and tendon sheaths. Jaffe et al. [1] regarded the synovium of the joint, tendon sheath, and the bursa as an anatomic unit that can give rise a common family of lesions, including localized and diffuse forms of pigmented villonodular synovitis (PVNS), giant cell tumor of the tendon sheath (nodular tenosynovitis), and the extra-articular pigmented villonodular synovitis arising from the bursa (pigmented villonodular bursitis (PVNB) or diffuse giant cell tumor of the tendon sheath). Involvement of bursa is the least common, and only few cases of exclusively extra-articular PVNB of the pes anserinus have been reported so far [1–11]. The purpose of this paper is to report an additional case of extra-articular pes anserine PVNB along with a review of literature. The patient was informed that data from the case would be submitted for publication, and she consented.

## 2. Case Report

**Figure 1:**
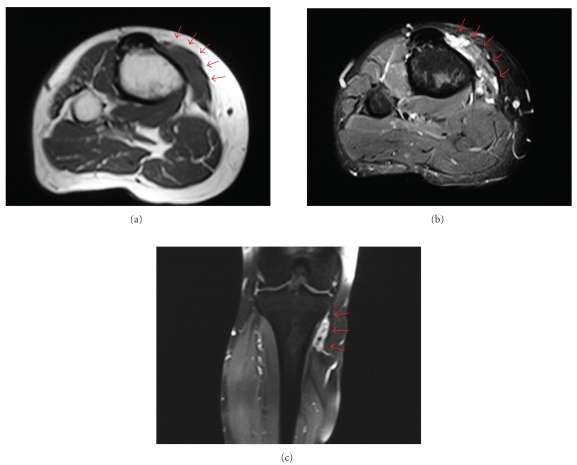
MRI with (a) axial T1 (TR 602, TE 40), (b) axial short tau inversion recovery (STIR; TR 4910, TE 40), and (c) contrast enhanced coronal T1 fat saturated(TR 540, TE 13) sequences showed a heterogeneous soft tissue mass at the insertion of pes anserinus conjoint tendon with peripheral and septal enhancement. The decreased interspersed signal on all images was due to hemosiderin deposits. There was no evidence of bone or joint involvement.

A 28-year-old female presented with a slowly enlarging intermittently painful mass over the medial aspect of her proximal right leg for the past three months. She denied any antecedent trauma. She denied any locking, clicking, loss of motion, or increased warmth around her knee. She denied any constitutional symptoms or any functional disability. On examination, there was a 4.0 × 3.0 cm relatively firm, tender mass on the medial aspect of the proximal leg at the level of the insertion of pes anserinus. The mass appeared fixed to the underlying tibia with no overlying warmth, erythema, induration, bruit, dilated vein, or significant regional lymphadenopathy. It was noncompressible with no change in size on limb elevation. Tinel's sign was negative, and distal neurovascular examination was unremarkable. There was full active painless range of motion at the knee without any instability or meniscal signs. The rest of her skeletal system examination was unremarkable. Radiographs were unremarkable. The magnetic resonance imaging (MRI) showed a 4.5 × 3.0 × 2.0 cm soft tissue mass at the insertion of pes anserinus conjoint tendon (Figures 1(a)–1(c)). There were areas of low to intermediate signals in all sequences, and the mass enhanced heterogeneously with contrast. There was no evidence of bone or joint involvement. Laboratory test results were noncontributory, including her coagulation profile.

**Figure 2:**
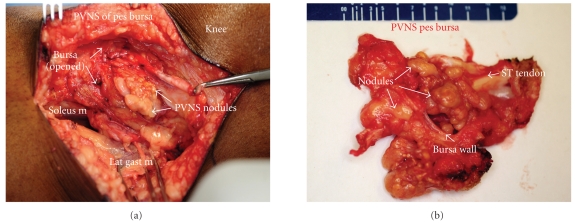
(a) Intraoperative photograph of the lesion and (b) the gross specimen showed multiple yellow to brown nodules inside the pes anserine bursa (ST: semitendinosus tendon).

**Figure 3:**
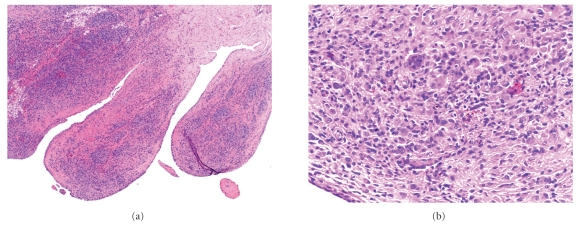
(a) Histopathological slide showed villous proliferation covered by reactive appearing synovial cells (H&E, ×4). The cores of the villi had a cellular infiltrate consisting of mononuclear cells and foamy histiocytes with scattered giant cells ((b); H&E, ×20).

An incisional biopsy was performed. The frozen sections showed it to be benign with multiple osteoclast like multinucleated giant cells in a mononuclear background, and a intralesional resection was performed. The specimen consisted of a brownish-yellowish lobulated multinodular mass in the pes anserinus bursa and measured 4.5 × 3.0 × 1.8 cm (Figures 2(a)–2(b)). The knee joint was not involved. Part of the semitendinosus tendon had to be sacrificed because of direct involvement of the lesion, and the remnants were tenodesed to the rest of the pes anserinus tendons. Histopathology showed osteoclast-like multinucleated giant cells in a mononuclear background with foci of hemosiderin deposits (Figures 3(a)–3(b)). There was no atypia, mitosis, or necrosis, and the surrounding soft tissue appeared unremarkable. A clinical diagnosis of PVNB of pes anserinus was made. The postoperative course was uneventful, and the patient is free of disease with no functional deficit at 2 years followup.

## 3. Discussion

Pigmented villonodular synovitis is a benign but potentially aggressive lesion, characterized by synovial villonodular proliferation with hemosiderin pigmentation and stromal infiltration of histiocytes and giant cells. Although it was suggested an inflammatory process [1], the insignificant degree of inflammation, the nodular growth pattern, propensity for local recurrence, distinct lack of changes characteristic of the lesion in the adjacent synovial tissue, and DNA flow cytometry point towards a neoplastic process [2, 12–15]. The incidence of PVNS is rare with an estimation of 1.8 patients per million population/per year [16]. PVNS is almost exclusively an intra-articular process. Extra-articular lesions are extremely rare and tend to occur in the same joint locations as the intra-articular PVNS, and are usually extensions from intraarticular lesions [17]. Rarer is the involvement of a true bursa (PVNB) with no articular communication. Although bursa around the knee (suprapatellar and popliteal) are more commonly reported, PVNB has also been sporadically described in the bursae around acromium, olecranon, iliopsoas, fingers, toes, temporomandibular region, and sacro-iliac areas [1, 14, 18–23]. A review of literature review revealed only twelve prior reports of exclusively extra-articular PVNB of the pes anserinus (Table 1) [1–11]. 

PVNB has shown a tendency to occur in young persons. Most of the cases are diagnosed prior to 40 years, with a mean age similar to PVNS [14]. Typically, symptoms are of relatively long duration and include pain and swelling, but limitation of knee range of motion is not common. The age range of PVNB of pes anserine has varied from 11–63 years with a mean of 31 years with no predilection for either sex or side [1–11]. Only one case was associated with a history of trauma 13 years previously [4]. Direct mechanical pressure to the bone and direct vascular foramina involvement was suggested as possible cause of bone involvement in one case [9]. PVNB, like PVNS, is usually monofocal, although Kay et al. reported a metachronous lesion in the wrist [7].

MRI is the imaging modality of choice and is helpful for diagnosis, surgical planning, and followup [5, 6, 8, 24]. The diffuse and nodular thickening of the synovium shows low to intermediate signal intensity, as compared to muscle signal, on all sequences. This low signal is due to the deposits of hemosiderin. Heterogeneous or septal enhancement may be observed after contrast administration. Apart from localization, MRI may also show the extension of abnormal synovial tissue in the periarticular region, in adjacent joints or bursae. Eckhardt and Hernandez [24] reported that PVNS was well visualized on both proton density-weighted images and gradient-echo images despite the blurring effect of hemosiderin-laden macrophages.

The clinical differential diagnosis includes simple bursal cyst, neurofibroma, fibroma of tendon sheath, or a hemangioma [1–11, 14]. A typical pes anserinus bursitis, meniscal, or ganglion cyst, may present with bursal fluid and characteristic MRI signal. Chronic bursitis may present as solid mass and then other forms of synovitis and neoplasm need to be considered. Apart from having a typical clinical picture, rheumatoid synovitis will not show giant cells, foam cells, or histiocytes but will have abundant lymphocytes. Hemosiderotic synovitis, such as chronic trauma and hemophilia, may be radiologically indistinguishable. However, the surface and subsynovial tissues are much richer in hemosiderin-filled macrophages and typically lack the giant cells and multitude of nonhemosiderin-filled macrophages that typify PVNS/PVNB [7]. A hemangioma may have calcified phleboliths and is morphologically homogenous with distinct histology [5, 6, 12]. A concomitant papillary synovium, with multiple telangiectasias mimicking intrasynovial hemangioma has been described by Kay et al. [7]. Histopathologically, the pronounced cellularity with a possibility of locally destructive mass can mislead to a diagnosis of malignancy [9, 14]. The geographical pattern of xanthomatous regions alternating with cellular hyalinized regions contrasts with the more uniform spindled appearance of synovial sarcoma and the primitive round cells of childhood rhabdomyosarcoma [14]. Moreover, cellular pleomorphism and mitotic activity goes in favor of a malignancy. Synovial sarcoma, usually, has biphasic morphology, may contain mucin, but shows no gross villi, nodules, or pigmentation. Moreover, about one-third of them may show mineralization. Diffuse giant cell tumors with prominent xanthomatous component must also be differentiated from inflammatory or xanthomatous forms of malignant fibrous histiocytoma or undifferentiated high-grade pleomorphic sarcoma [14]. The latter usually occur in retroperitoneum and contain xanthomatous areas and spindled areas resembling the conventional form. Moreover, these have predominant acute inflammatory background, which contrasts with the modest number of chronic inflammatory cells in giant cell tumors. Focal necrosis may also be present in PVNS due to torsion of a pedunculated nodule. The synovium-based location and apparent maturation at the periphery may be helpful in diagnosis. Because of this wide spectrum of differential diagnosis, a histologic diagnosis becomes essential before planning the definite surgical procedure (intralesional, marginal, or wide local resection).

Although much has been written about behavior and treatment of PVNS, the literature is sparse on PVNB. Recurrence has been reported to be as high as 40–50% for diffuse PVNS and most of them have been correlated with a location in the knee and incomplete excision [14]. Morphology has not been found to be predictive of recurrence. Although the followup has been limited on most of the reported cases (Table 1), only one had recurred 13 years later. This could be attributed to the localized and extra-articular nature of pes anserinus PVNB, which allows the surgeon to be more aggressive and completely resect the mass without fear of significant functional loss.

## 4. Conclusion

Extra-articular PVNB of the pes anserinus is a rare cause of medial knee pain and localized swelling about the proximal tibia. Awareness of this rare presentation is important because it will help in correct diagnosis and avoid overtreatment. An MRI is useful to delineate the soft tissue extent and bone involvement, and exhibits areas of low signal intensity on all sequences due to hemosiderin deposits. Marginal or intralesional resection of the lesion seems to provide excellent clinical results with no need for adjuvant modalities like radiotherapy. As with other rare lesions, clinical and radiographic findings in addition to histological examination are essential for correct diagnosis.

## Figures and Tables

**Table 1:** Giant cell tumor of the pes anserine bursa (extra-articular pigmented villonodular bursitis): a review of literature.

Authors/year	Age (years)/ gender/side	Clinical presentation	Size	Treatment	Outcome
Jaffe et al., 1941 [1]	34/NS	Mass more than 3 years, but no limitation of range of motion	>5-6 cm	NS	NS

Granowitz et al., 1976 [3]	NS	NS	NS	NS	NS

Present et al., 1986 [9]	54/M/R	Pain with moderate joint effusion but a palpable mass × 4 years. There was a lytic lesion in the tibia	NS	Arthroscopy, followed by incisional biopsy, resection of mass and curetting and cementing of tibial lesion	NS

Jelinek et al., 1989 and 1994 [5, 6]	NS/L, 20/F/L	NS	NS	NS	NS

Kay et al., 1996 [7]	11/M/R	Swelling without limitation of range of motion × 18 months. Also had a metachronous lesion in the wrist about 18 months prior	3.5 × 8.0 cm	Open biopsy, followed by wide resection and tenodesis of involved tendons	DF × 12 months

Abdelwahab et al., 2002 [2]	63/F/R	Increasing swelling and recent-onset pain with limitation of range of motion × 2 years. Also had associated Sjogren's syndrome	5.0 × 3.5 × 2.0 cm	Resection of entire mass with repair of resected tendon	DF × 18 months

Sami et al., 2003 [11]	26/F/L	Worsening pain and swelling with no limitation of range of motion × 2 months	4.5 × 3.0 × 2.5 cm	Open biopsy, followed by marginal excision	DF × 27 months

Maheshwari et al., 2007 [8]	17/F/L	Slowly enlarging mass × 3 years	5.6 × 3.8 × 3.5 cm	Open biopsy, followed by marginal resection	DF × 2 years
	18/M/L	Pain with jogging × 2 years	6.1 × 3.5 × 3.4 cm		DF × 18 years

Riccio et al., 2007 [10]	36/M/L	Swelling and pain with jogging × 1 month	3.5 × 6.5 cm	Marginal resection	DF × 30 months

Hepp et al., 2008 [4]	28/F/L	Swelling and pain × 4 weeks. History of trauma 13 years previously with excision of a similar mass (histologically read as PVNS) in the same location	4.0 × 4.0 × 3.0 cm	En bloc resection	NS

NS: not specified; DF: disease free; PVNS: pigmented villonodular synovitis.
